# Alternative Methods of Vaccine Delivery: An Overview of Edible and Intradermal Vaccines

**DOI:** 10.1155/2019/8303648

**Published:** 2019-03-04

**Authors:** E. Criscuolo, V. Caputo, R. A. Diotti, G. A. Sautto, G. A. Kirchenbaum, N. Clementi

**Affiliations:** ^1^Microbiology and Virology Unit, “Vita-Salute San Raffaele” University, Milan, Italy; ^2^Pomona Ricerca S.r.l., Turin, Italy; ^3^Center for Vaccines and Immunology, University of Georgia, Athens, GA, USA; ^4^Cellular Technology Ltd., Shaker Heights, OH, USA

## Abstract

Vaccines are recognized worldwide as one of the most important tools for combating infectious diseases. Despite the tremendous value conferred by currently available vaccines toward public health, the implementation of additional vaccine platforms is also of key importance. In fact, currently available vaccines possess shortcomings, such as inefficient triggering of a cell-mediated immune response and the lack of protective mucosal immunity. In this regard, recent work has been focused on vaccine delivery systems, as an alternative to injectable vaccines, to increase antigen stability and improve overall immunogenicity. In particular, novel strategies based on edible or intradermal vaccine formulations have been demonstrated to trigger both a systemic and mucosal immune response. These novel vaccination delivery systems offer several advantages over the injectable preparations including self-administration, reduced cost, stability, and elimination of a cold chain. In this review, the latest findings and accomplishments regarding edible and intradermal vaccines are described in the context of the system used for immunogen expression, their molecular features and capacity to induce a protective systemic and mucosal response.

## 1. Introduction

One of the ten greatest public health achievements of the last century was preventative vaccination [1]. Vaccines have successfully reduced the spread of diseases and mitigated mortality associated with infectious agents such as diphtheria, tetanus, polio, measles, mumps, rubella, and hepatitis B [2]. In spite of the many successes achieved by vaccines, novel technologies and administration routes remain one of the main focuses in the vaccinology field. Although many licensed vaccines are administered by injection, in certain cases, this administration route suffers from limitations. In particular, injectable vaccines require trained personnel for the administration of the vaccine and may require multiple doses or inclusion of an adjuvant. Moreover, injectable vaccines may require specialized storage and transport conditions. From an immunological point of view, injectable vaccines are capable of eliciting robust systemic humoral responses while conferring weaker T cell-mediated immunity and mucosal protection [3, 4]. Importantly, T cell effector activity and mucosal immunity both contribute to prevention and control of infection from pathogens targeting the mucosa [5].

To improve on this limitation, alternative vaccine delivery methods coupled with novel formulations and production systems have recently been proposed. Numerous studies have focused on vaccines delivered to the mucosal interface or intradermally, demonstrating rapid and wide biodistribution of the antigen and the capacity to induce both protective mucosal (mainly mediated by secretory IgA [SIgA]) and systemic cellular and humoral responses [6–8].

In this review, we discuss current advances and advantages of edible systems based on plants, algae, yeast, insect cells, and lactic acid bacteria and of the intradermal immunization route.

### 1.1. The Mucosal Delivery and the Immune Response

The efficacy of the mucosal administration route is largely based on the fact that mucous membranes constitute the largest immunologic organ in the body. Moreover, this interface is endowed with well-organized lymphatic structures, termed mucosa-associated lymphoid tissue (MALT), containing both the innate and adaptive (T and B cells) arms of the immune system [9]. Furthermore, antigen-specific SIgA plays a pivotal role in protecting mucosal surfaces from both microbe adhesion and toxin activities [8]. Thus, the development of novel vaccine delivery platforms implementing the elicitation of pathogen- or toxin-specific SIgA, as well as systemic IgG, is pivotal to improve vaccine effectiveness [10].

**Figure 1:**
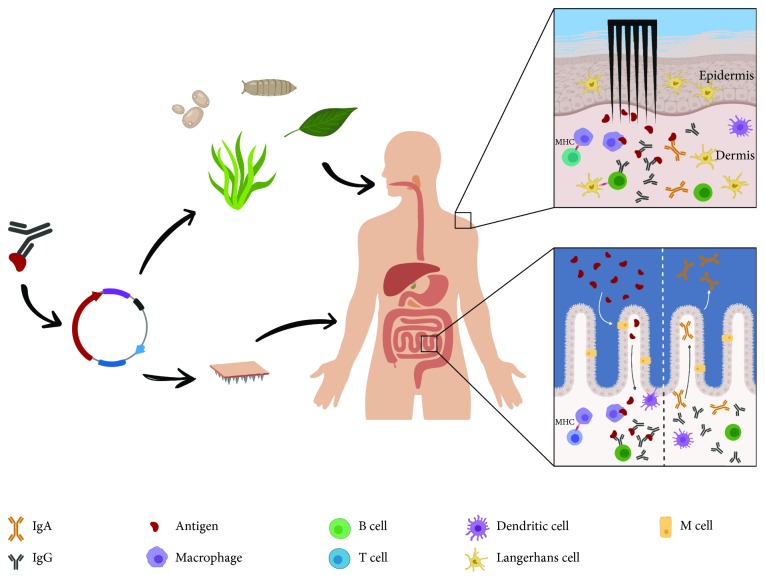
Alternative methods of vaccine delivery. Development of rationally designed vaccines starts with the identification of the gene encoding for the protective antigenic protein(s). Subsequently, the antigen(s) can be incorporated into different edible systems, as plants, algae, insects, or yeasts, or used for intradermal formulations to induce a mucosal protective response. Following the administration of the edible vaccine and the subsequent passage of the antigen(s) through the M cell compartment delivering it to dendritic cells, the individual's immune system triggers a response leading also to specific IgA production and secretion. Similarly, patches with coated microprojections or biodegradable needles activate Langerhans cells and dermal dendritic cells in the skin dermis. These cells capture and present the antigen(s) to T and B lymphocytes, triggering both a mucosal and a systemic immunity.

To date, the most well-studied vaccine delivery platforms capable of eliciting both mucosal and systemic immunities are edible or intradermal vaccine formulations (Figure 1). Oral vaccines stimulate the generation of immunity in gut-associated lymphoid tissue (GALT), which includes lymph nodes, Peyer's patches (in which lymphocytes are the major component: ~75% are B cells, while ~20% are T cells), and isolated lymphoid follicles in the gastrointestinal tract (GIT). An effective immunization using oral vaccines is achieved when sufficient quantities of antigen are transported across the mucosal barrier by M cells into Peyer's patches and subsequently presented to T cells by antigen-presenting cells (APCs) [11]. Briefly, professional APCs display peptide fragments of the antigen in the context of the major histocompatibility complex (MHC) on their surface, which leads to activation of CD4^+^ T cells [12]. Subsequently, activated CD4^+^ T cells support germinal center development, including B cell affinity maturation and class switching to IgA, through providing CD40/CD40 ligand interactions and cytokine secretion [13–15]. Moreover, through the expression of specific chemokine homing receptors (e.g., CXCR5 or CCR10), antigen-experienced B cells migrate to distant effector regions where they differentiate into plasma cells capable of secreting dimeric or polymeric IgA molecules that are transported into the intestinal lumen as SIgA [10, 16].

In the context of edible vaccines aimed at eliciting pathogen-specific responses, it will be necessary to overcome mucosal tolerance. Briefly, mucosal tolerance is achieved against certain foreign antigens, such as those contained in our food, and serves to prevent unnecessary and potentially detrimental immune responses in the gut mucosa. Due to this phenomenon, an erroneous mucosal vaccine formulation could induce a T_reg_-based tolerogenic response instead of Th17-mediated protective immunity [17]. This potential shortcoming can be circumvented using several strategies, including incorporation of an appropriate adjuvant in the vaccine formulation or using sufficiently high doses of antigen to promote induction of effector rather than regulatory cells [5, 11]. Moreover, in the context of edible-based vaccine immunizations, it will also be important to consider the characteristics of the GIT, in which several factors, including proteolytic enzymes, acidic pH, bile salts, and limited permeability, may hinder the induction of a protective immune response [10]. To this end, conjugation of the vaccine antigen with specific ligands that enhance their uptake by M cells represents a focus of ongoing studies aimed at improving immunogenicity [18]. Moreover, antigen bioencapsulation avoids degradation and conformational alterations [19].

### 1.2. Overview of Edible Vaccines

In the following sections, we review the various strategies underlying the development of edible vaccines. In particular, we focused on plant, algae, insect cells, whole yeast, and lactic acid bacteria-based vaccines and describe the advantages and limitations of individual systems.

### 1.3. Plant-Based Vaccines

Plants have been extensively used for developing novel biopharmaceutical-producing platforms in recent years, as they promote proper folding of exogenous proteins and are economically sustainable [20, 21]. This is also known in the context of “molecular farming,” in which biomolecules of commercial value are produced in genetically engineered plants. There are several ongoing clinical trials using purified antigens transiently produced in tobacco plants (*Nicotiana benthamiana*) for injectable vaccine formulations. For example, Medicago recently completed a phase II clinical trial using a plant-derived, virus-like particle (VLP) quadrivalent influenza vaccine and announced a phase III clinical study in the last year (ClinicalTrials.gov identifier: NCT03301051) [22].

Owing to the fact that plants are edible, the notion that they could serve as a delivery vehicle, as well as biofactories, led to their use for oral vaccination in the early 1990s [23]. In recent years, additional studies have sought to overcome the limitations of conventional vaccines through development of edible formulations [24, 25]. Since the inception of the idea, it has been evident that using plants to produce vaccines would have several advantages. First, plant vaccines would likely have a low production cost and could be easily scaled-up, as has been demonstrated by the biopharmaceutical industry. Molecular farming gained visibility thanks to the success of ZMapp, the experimental drug against the Ebola virus that was produced in *Nicotiana* plants [26]. However, unlike biomolecule production, edible vaccine formulations do not need processing or purification steps before administration, which serves to further lower production-associated costs. Indeed, another advantage of this strategy is that plant cells would provide antigen protection due to their rigid cell wall. This is also known as the bioencapsulation effect and could increase bioavailability of antigenic molecules to the GALTs through preserving structural integrity of vaccine components through the stomach to elicit both a mucosal and a systemic immune response. Additional strategies for antigen protection can be achieved through targeting biomolecule expression inside chloroplasts or other storage organelles [27] or in the protein bodies of seeds [28, 29]. This technology also offers advantages in terms of storage and cold chain-free delivery due to the high stability of the expressed antigens bioencapsulated within the plant and seed tissues. Moreover, plant materials can be stored at elevated temperatures for longer periods and grown where needed, making this strategy even more attractive for developing countries [30]. Finally, plant-based oral vaccines are characterized by improved safety relative to traditional recombinant vaccine platforms, especially since contamination from mammalian-specific pathogens can be eliminated [30]. Indeed, some studies using lyophilized leaves have shown their advantages over fresh materials such as long-term stability, higher antigen content, and lower microbial contamination. As an example, freeze-dried CTB-EX4-expressing (CTB: cholera toxin B subunit; EX4: exendin-4) leaves were shown to be stable for up to 10 months at room temperature, and lettuces expressing soluble antigen (PA; protective antigen from *Bacillus anthracis*) were successfully stored for up to 15 months at room temperature without showing antigen degradation [31]. The antigen content in lyophilized leaf materials was also 24-fold higher than fresh leaves. An additional benefit of lyophilization was its ability to remove microbial contamination. While lyophilized lettuce had no detectable microbes, fresh leaves contained up to approximately 6000 cfu/g microbes when plated on various growing media [31].

To date, vaccine antigens have been transformed into many edible species including lettuce, tomato, potato, papaya, carrot, quinoa, and tobacco [32]. Their proper folding and enhanced expression have also been tested in animal models, proving the immunogenicity of antigens produced in these systems [24, 33].

To obtain high quantities of the protein of interest, both nuclear and chloroplast genomes have been successfully engineered. However, the latter option is preferred owing to some advantages including high levels of transgene expression (up to 70% of total soluble proteins (TSP)) [34, 35], bioencapsulation effect, and regulatory concerns since transgene containment is assured by the fact that plastids are not spread via pollen in most plants. Moreover, incorporation of vaccine antigens into the chloroplast genome would also enable the expression of multiple genes in a single operon, which would be very attractive for multivalent vaccine development. Likewise, this approach may enable the production of vaccines conferring protection against multiple infectious agents and would serve to further reduce costs associated with vaccine production and administration [36].

Unfortunately, there are some disadvantages undermining their applications. First, plastids are not suitable for production of antigens that require glycosylation for proper folding or those antigens in which a protective immune response requires glycan recognition. However, nuclear transformation represents a valid option. Secondly, antigen expression can be either transient or stable in plants, but the second is preferred in order to obtain a stable genetic resource. In fact, transgenic seeds represent a constant resource to grow the transgenic plants and to extract proteins. However, stable transformation is time-consuming [25]. Moreover, expression in stable transformed crop plants suffers from low yields, typically less than 1% of TSP [36]. On the other hand, transient expression technology using either *Agrobacterium* or viral vectors is robust, quick, and easy to manipulate [37]. However, this expression is typically unstable [30]. Another important challenge of plant-based oral vaccines is the lack of a proper dosing strategy because low doses may not be able to induce a sufficient immune response and high doses, as previously described, may lead to immune tolerance. To this end, freeze-drying methods were implemented to stabilize plant biomass, concentrate the antigen, and achieve an accurate dosage by quantifying the antigen in terms of dry biomass weight, as mentioned above [31, 38].

To date, there are some plant-based vaccines for the hepatitis B virus (HBV), rabies virus, Norwalk virus, enterotoxigenic *E. coli*, and *Vibrio cholerae* in phase 1 clinical trials (Table 1). Many others are still in preclinical development, including vaccines targeting a variety of pathogens such as avian influenza viruses (HPAI H5N1) [39], *Helicobacter pylori* [40], and coronaviruses [41].

### 1.4. Algae-Based Vaccines

Green microalgae, such as *Chlamydomonas reinhardtii*, represent another viable option for vaccine production. However, some disadvantages of plant-derived vaccines, such as low expression levels and improper glycosylation of antigen proteins, have been described [52]. Thus far, only chloroplast transformation is possible [52], and only a single organelle is present, even if it occupies half of the cell volume [53].

Stable transformed lines of green algae are easy to obtain and can lead to increased yield of expressed antigens. In fact, unicellular green algae have all the positive characteristics of plant systems, plus unique advantages over terrestrial plants. Biomass accumulation is extremely fast and can be used in its entirety. Their growth neither has seasonal constraints nor relies on soil fertility. Cross-contamination of nearby crops cannot occur, as algae can be cultured with enclosed bioreactors [54]. Furthermore, in regard to regulatory aspects, green algae, such as *C. reinhardtii*, are generally recognized as safe (GRAS) by the FDA. Finally, algae can be easily lyophilized and, when dried, can be stored at room temperature for up to 20 months without losing antigenic efficacy [55]. In fact, the algae cell wall assures the bioencapsulation effect, as it was proven to prevent antigen degradation by enzymes of the GIT [55].

Collectively, these characteristics indicate that algae would be an ideal host for vaccine transport without a cold chain supply. Thus, as already described for plant-derived edible vaccines, the low cost and simpler logistic in terms of manufacturing, storage, delivery, and administration of the algae-based technology make it an ideal system in the context of resource-limited settings compared to conventional vaccine formulations.

There are no algae-based vaccines currently in clinical trials; however, preclinical formulations against human papillomavirus (HPV), HBV, and foot-and-mouth disease virus (FMDV) are under development [32, 52, 56] to overcome some technical problems, such as a low expression level from the nuclear genome and lack of glycosylation following chloroplast expression [52].

### 1.5. Insect Cell-Based Vaccines

Insect cell systems have been widely adopted because of their capacity to produce high levels of proteins and to perform cotranslational and posttranslational modifications, including glycosylation, phosphorylation, and protein processing. This expression platform allows for generation of stable transformed cell lines or transient expression driven by recombinant baculovirus infection. The baculovirus-insect cell expression system, referred to as BEVS, is one of the most well-known and used systems for large-scale production of complex proteins and, most recently, for the development of subunit vaccines [57]. To date, there are three commercially available vaccines produced in insect cells for different indications: Cervarix (GSK) for cervical cancer, Flublok (Protein Sciences, now owned by Sanofi Pasteur) for influenza, and PROVENGE (Dendreon) for prostate cancer [58].

Importantly, the baculovirus expression system is not limited only to cultured cells. Insect larvae or pupae can be used for protein production. In the context of edible vaccines using insect larvae or pupae, silkworm *Bombyx mori* larvae or pupae have been commercially used for the production of recombinant proteins and also as a feasible delivery system for the vaccine [59, 60]. As mentioned above, the baculovirus-silkworm expression system is able to perform cotranslational and posttranslational modifications and also able to produce large amount and multiple proteins. Moreover, since baculovirus is unable to replicate in vertebral animals, it can be considered a GRAS. Furthermore, the presence of protease inhibitors and biocapsule-like fat in the silkworms may protect recombinant proteins from enzymatic digestion in the GIT [23, 61].

Several vaccine prototypes are currently under evaluation, and strong systemic immune protective responses support the use of silkworm as a mucosal vaccine vector, as shown, for example, by high immunogenicity in mice of the urease B subunit of *Helicobacter pylori* produced in silkworm [60, 62]. While the data collected so far support the possible use of baculovirus-silkworm vaccines as a promising edible vaccine platform, it is only approved for food ingestion in a few Asian countries.

### 1.6. Whole-Cell Yeast-Based Vaccines

The industrial usage of yeasts cells for production of heterologous proteins has been well described [63, 64]. Additionally, the capability of this system to perform posttranslational modifications, the GRAS status, and the cellular wall that could protect the antigen across the GIT make engineered yeasts an attractive vaccine delivery system [23, 65]. In addition, the major drawback of this system is hyperglycosylation of recombinant proteins, but it has been already addressed by generating defective N-glycosylation strains of yeasts [66, 67].

Whole-cell yeast-based vaccines have been studied for their ability to elicit an immune response. Remarkably, some preclinical studies based on orally administrated *Saccharomyces cerevisiae* and developed for different infectious agents, such as HPV and *Actinobacillus pleuropneumoniae*, showed that this delivery system is able to induce a protective mucosal and a systemic immune response [68–70].

Moreover, the increased immunogenicity of this delivery system could be explained by the adjuvant activity of *β*-glucans on the yeast cell wall, which demonstrates immunomodulatory and adjuvant effects through binding of innate pathogen receptors on macrophages, DC, and neutrophils [71]. Currently, two clinical trials have been developed: GS-4774 for HBV treatment and GI-5005 for hepatitis C virus (HCV) treatment (Table 2). Regarding the clinical trial for GS-4774, despite the positive results obtained from phase 1 [72], the second phase, conducted in virally suppressed, noncirrhotic patients with chronic HBV infection did not show a clinical benefit. However, other safety and efficacy studies have been conducted on another group of patients (in particular, in treatment-naïve patients with chronic HBV) [73]. Regarding the clinical trial for GI-5005, phases I and II reported promising results [74]. In particular, in this trial, GI-5005 was used also in combination with Peg-IFN/ribavirin. However, data on efficacy have not been published yet.

### 1.7. Lactic Acid Bacteria-Based Vaccines

Lactic acid bacteria (LAB) are Gram-positive, nonsporulating, and nonpathogenic bacteria that have been used for decades for the production and preservation of food as well as for therapeutic heterologous gene expression, like the production of different anti-human immunodeficiency virus (anti-HIV) antibodies (scFV-m9, dAb-m36, and dAb-m36.4) by *Lactobacillus jensenii* and the production and functional expression of the antilisterial bacteriocin EntA in *L. casei* [75–77]. Given these and the ability of LAB to elicit a specific immune response against recombinant foreign antigens, these bacteria have been considered potential candidates as mucosal vaccine vectors. This delivery system can confer protection against antigen degradation and, thanks to its uptake at the GIT level, can activate both innate and adaptive immune responses [78, 79].

Many LAB, in particular, *Lactobacillus spp* and *Bacillus subtilis*, were used in preclinical studies against different infectious diseases. Different results have been obtained from these studies, but an elicited immune response was observed in all of them. As an example, the production of high levels of specific IgA and systemic IgG after immunization with *bacillus* spores expressing toxin A peptide repeat was reported [80], while in another paper, *L. lactis* expressing HEV antigen ORF2 vaccine was tested and a specific Th2-based cell-mediated immune response was revealed as well as the production of specific mucosal IgA and serum IgG [81]. Another study reported a Th1/Th2 immune response elicited after the immunization with Csenolase-expressing *Bacillus subtilis* [82]. Another example is the oral administration of *B. subtilis* spores expressing urease B of *Helicobacter pylori* that provide protection against *Helicobacter* infection [83].

An important feature of LAB is their natural adjuvanticity and their immunomodulatory effects, although the molecular mechanism of these capabilities is not completely understood [84]. Moreover, other studies reported an effect on dendritic cell maturation and an induction of cytokine secretion [85, 86]. Despite the promising characteristics of recombinant LAB as mucosal vaccine vectors and given the encouraging results from murine studies, some aspects need to be taken into consideration, namely, the fact that vaccine strains cannot be considered avirulent, even if it could be listed as GRAS, due to potential transfer of antibiotic selection markers among microbes [78, 87]. Furthermore, other factors are important for the development of LAB-based vaccines. As an example, the necessity of a suitable delivery system since different administration routes produce different immune effects. Additionally, the role of specific adjuvants and the correct localization (intracellularly or on the bacterial surface) of each expressed antigen need consideration [88]. Overall, additional studies and clinical trials are needed for the development of efficient vaccines based on LAB.

A different carrier system based on nonrecombinant *Lactococcus lactis* bacteria was recently developed. This system, called Gram-positive enhancer matrix (GEM), is composed of the rigid peptidoglycan (PGN) cell wall of the bacterium resulting in a nonliving particle that preserves the shape and the size as the original bacterium [89]. GEMs are used in two different ways: mixed with vaccine antigens as adjuvants or as antigen protein carriers, with the antigens bound to the surface of GEMs.

Regarding the use of GEMs as adjuvants, because of their nature, GEMs are safer adjuvants compared to others. Moreover, they retain the inflammatory properties of live bacteria and enhanced specific mucosal and systemic immune responses of the influenza subunit vaccine [90–92]. Therefore, the use of GEMs was further examined in a study investigating the immune response elicited by intranasal delivery of the influenza subunit vaccine coadministrated with GEM (FluGEM). In detail, an influenza-specific memory B cell response and the presence of long-lived antibody-secreting plasma cells were reported. Additionally, this immune response was able to confer protection from influenza infections [91]. These important results obtained in murine studies have led to a phase I clinical trial which confirmed the positive preclinical data. Systemic hemagglutination inhibition (HAI) titers and local SIgA responses were reported. Further studies will assess if this immune response confers protection against the influenza virus [93].

GEMs have also been used as antigen protein carriers. In particular, antigens are bound to GEM through the presence of a PGN-binding tag (Protan) in the antigen. Several works used this vaccination strategy, and the data support the potential of GEMs as safe vaccine delivery vehicles and their ability to elicit systemic antibodies [94–97]. Moreover, GEMs are also able to present several antigens at the same time which could be helpful for the preparation of multivalent vaccines [98]. Furthermore, the delivery of an adjuvant (GEMs) and an antigen together has been correlated with enhanced vaccine immunogenicity [97]. Lastly, as opposed to a vaccine based on LAB, the absence of recombinant DNA avoids its dissemination into the environment. However, the inability of GEMs to colonize any compartment does not allow the reduction of the number of vaccine doses.

These promising premises allowed the development of a vaccine against respiratory syncytial virus (RSV). In particular, an intranasal formulation based on the trimeric RSV fusion protein coupled with GEMs and named SynGEM was developed. Also, in this case, positive results from studies in mice and rats have been obtained, and as for FluGEM, vaccination with SynGEM resulted in the induction of a robust systemic and mucosal immune response as well as a balanced cytokine profile. These data supported further study of this vaccine in phase I clinical trial, which is currently ongoing [97]. In conclusion, GEMs represent an interesting vaccination strategy either as adjuvant or as antigen protein carriers, but as in the case of vaccine based on LAB, further studies are needed.

### 1.8. The Intradermal Vaccine Delivery and Its Associated Immune Response

Another vaccine delivery route capable of triggering both systemic and mucosal immunities is the intradermal route, in which the antigen is delivered through the skin using recently developed self-administrable devices. In particular, the application of microneedle technology overcomes the skin permeation barrier imposed by the stratum corneum and facilitates antigen delivery. The efficacy of this new microneedle-based immunization approach is due to the presence of several types of immune cells (such as DCs, T lymphocytes, NK cells, macrophages, and mast cells) in the epithelium [99, 100]. In fact, the cells that are responsible for triggering the inflammation cascade in the skin are the Langerhans cells (comprising 2-4% of epithelial cells). Langerhans cells are a specific DC subset that migrates into the lymph node following antigen capture and aids in the initiation of an adaptive immune response [101]. These cells are also efficiently stimulated by pathogen-associated molecular patterns (PAMPs) using an array of germline-encoded pattern recognition receptors (PRR), including toll-like receptors (TLR) and langerin (CD207) [100]. Importantly, skin resident mast cells are also key drivers of the innate immune response in the skin through the release of granules containing inflammatory mediators [102].

### 1.9. Intradermal Vaccination

Using conventional syringes for intramuscular or subcutaneous vaccinations, large volumes of vaccine solution can be injected (≥1 mL). Thus, the choice of the muscle or hypodermis as vaccination targets is mainly based on convenience [99]. Intradermal immunization for vaccine delivery is an upcoming strategy showing significant advantages over conventional vaccination routes. In particular, in the last few years, intradermal vaccination has gained momentum as an alternative and more effective vaccine delivery route, both from a scientific and a commercial point of view (Table 3).

Intradermal vaccination designates the delivery of an antigen directly into the dermis with a syringe, a needle, a microneedle, or a pressure injector. The standard intradermal immunization technique was invented by the French physician Charles Mantoux in 1910, while he was developing the tuberculin test. This technique allows the injection of 100-200 *μ*L of vaccine solution. However, Mantoux's technique requires skilled medical personnel to be performed [103]. Recent advancements have led to the development of techniques and instruments that can overcome the difficulties associated with intradermal administration [104]. In fact, different devices have been developed over the years for intradermal vaccination. Among them, solid microneedles, particle injectors, and self-administrable patches with coated microprojections or biodegradable needles have been described [105]. As previously mentioned, intradermal vaccination can induce mucosal and systemic immunities. These immunological capabilities, coupled with its ease of access, make the intradermal route an attractive vaccination delivery target [106].

Intradermal vaccination has been demonstrated to be very safe. In fact, novel devices involve the use of needles with a smaller size than the usual (25 *μ*m and 1 mm) and make it possible to bypass the corneous layer of epidermis by creating transient micropores in the cutaneous tissues. Even if some studies have shown that intradermal vaccination can be associated with a higher incidence of local reactogenicity, including primarily mild pain, swelling, and redness, systemic side effects have not been reported. In fact, the intradermal route limits the transfer of vaccine components to the blood circulation (and the risk of septic shock) and the possible toxicity due to hepatic first-pass effect [107]. Typically, when present, local effects resolve quickly, as reported in a study comparing the safety and immunogenicity of a large number of intradermal versus intramuscular influenza vaccines [108].

Another important aspect is the possibility of improving the immunogenicity of various vaccines in immunocompromised hosts as well as during pregnancy via the intradermal route [109, 110]. As an example, it has been reported that the HBV vaccine fails to yield seroconversion in 3-5% of recipients. However, a significant improvement was observed following intradermal vaccination [111]. Additionally, it has been demonstrated that in patients on dialysis or in HIV-positive subjects, the intradermal route was more immunogenic than the standard intramuscular route [112].

From a commercial point of view, intradermal vaccination has been primarily explored for its ability to elicit equivalent antibody responses at lower doses, a phenomenon typically described as “dose sparing” [113]. In this regard, the advantage of a low dose is most evident in high-surge situations, such as during pandemic and seasonal influenza waves, in which large populations are at an increased risk and large amounts of new antigen preparations are quickly required each year [114–116]. Above all, dose sparing is also important in a large-scale setting and in reducing the production-associated costs, especially in developing countries, where the price of the vaccine limits its use and coverage. In this regard, the U.S. Food and Drug Administration (FDA) approved the trivalent inactivated intradermal influenza vaccine for use in adults 18-64 years of age for use during the 2012-2013 season, and a quadrivalent formulation was subsequently approved in 2014. However, similar to intramuscular vaccines, the formulation of these approved intradermal vaccines is liquid and thus still dependent on the cold chain and administered through a syringe. For these reasons, novel dried solid microneedle devices, while eliciting comparable immunogenicity to intramuscular-administered vaccines, represent an innovative approach to facilitate self-administration and allow vaccine storage at room temperature [117].

## 2. Conclusions

Infectious diseases represent a global concern, and the most effective strategy to reduce them is vaccination. Unfortunately, not every disease can currently be prevented through vaccines. However, many strategies have been developed against infectious agents, such as the generation of neutralizing antibodies, antibiotics, and antiviral drugs [124–130], and innovative approaches are currently under development [131–133].

Many vaccines have been developed and approved against various pathogens, and countless studies have been conducted to improve their efficacy by testing new adjuvants and performing the rational identification of the antigen formulations and pathogen contaminations [134–136]. Promising results have been also achieved by changing the delivery strategy. In fact, most of the approved vaccines are administrated by injection with intrinsic limitations like those concerning the immunological aspect. Injected vaccines are able to elicit a strong humoral immunity but a weak cellular response. In addition, this type of administration is strongly associated with a systemic immunity but with a lack of mucosal response, which is helpful to block the early stages of infection since most pathogens infect through the mucosal membranes.

For these reasons, new vaccination strategies have been proposed. In particular, edible vaccines, triggering the GALT, and intradermal approaches, involving Langerhans cells, are able to elicit both a mucosal and a systemic immune response. The increased knowledge of these approaches has led to the progression of many preclinical studies and several promising clinical trials (Tables 1, 2, and 3). Moreover, these vaccine strategies are considered safe and cost-effective as no extensive antigen processing is needed [137, 138] and they are easy to administrate (Table 4). In fact, due to the opportunity of self-administration and ease of distribution compared to an injection-based approach, these two vaccination systems could improve the overall coverage.

There remain a number of obstacles and drawbacks associated with each antigen delivery platform that still need to be addressed (Table 4). Presently there are no FDA-approved compounds for edible vaccination, but new emerging technologies like nanoparticles (NPs) could help to control antigen bioavailability to avoid mucosal tolerance. NPs are particles with a mean size of 10-100 nm (up to 2000 nm), which can be used as carriers and/or adjuvants in vaccine preparation [139–141]. Moreover, NPs can be targeted to specific cell populations. As an example, NPs can be coated with antibodies recognizing a surface protein on dendritic cells [142, 143]. This approach enabled a more accurate measurement of the quantity of antigen required to elicit an immune response [144]. Finally, a more efficient immunization was demonstrated using NP-based approaches combined with an intradermal vaccine delivery [145], while oral delivery needed further investigations as they have been tested only *in vitro* [146, 147].

In conclusion, novel approaches eliciting a stronger mucosal response are showing promising results both in preclinical and clinical studies. Further studies are needed to implement and improve these delivery systems; however, mucosal delivery is becoming the most preferred mode of vaccination.

## Figures and Tables

**Table 1:** Status of development of plant-based vaccines.

Pathogen	Antigen	Plant host	Expression system	Indication	Route of administration	Clinical trial status	Clinical trial ID	Refs
Enterotoxigenic *E. coli*	LT-B	Potato	Transgenic	Diarrhea	Edible	Early phase 1	▶	[42]
Enterotoxigenic *E. coli*	LT-B	Maize	Transgenic	Diarrhea	Edible	Early phase 1	▶	[43]
Norwalk virus	CP	Potato	Transgenic	Diarrhea	Edible	Early phase 1	▶	[44]
Rabies virus	GP/NP (fusion)	Spinach	Viral vector (transient)	Rabies	Edible	Early phase 1	▶	[45]
HBV	HBsAg	Lettuce	Transgenic	Hepatitis B	Edible	Early phase 1	▶	[46]
HBV	HBsAg	Potato	Transgenic	Hepatitis B	Edible	Phase 1	NCT01292421	[47]
*Vibrio cholerae*	CTB	Rice	Transgenic	Cholera	Edible	Phase 1	UMIN000009688	[48–51]

HBsAg: hepatitis B surface antigen; CP: capsid protein; GP: glycoprotein; NP: nucleoprotein; CTB: cholera toxin subunit B. ▶: restricted cohort study design.

**Table 2:** Status of development of whole yeast-based vaccines.

Pathogen	Antigen	Yeast host	Expression system	Indication	Clinical trial status	Clinical trial ID	Refs
HBV	HBV (X/S/core)	*Saccharomyces cerevisiae*	Stable	Chronic HBV	Phase 2	NCT01943799	[73]
						NCT02174276	
HCV	HCV (NS3/core)	*Saccharomyces cerevisiae*	Stable	Chronic HCV	Phase 2	NCT00606086	[74]

X: hepatitis B regulatory protein; S: hepatitis B surface antigen; NS3: hepatitis C nonstructural protein.

**Table 3:** Status of development of some intradermal vaccines.

Pathogen	Formulation/antigen	Indication	Clinical trial status	Clinical trial ID	Refs
Influenza virus	Split virus	Influenzas A and B	Approved	NCT01712984, NCT02563093, NCT02258334, NCT01946438	[118]
Enterotoxigenic *E. coli*	dmLT^∗^	Gastroenteritis	Phase 1	NCT02531685	[119]
HBV	HBsAg	Hepatitis B	Phase 1	NCT02186977	[120]
Dengue virus	Attenuated virus	Dengue fever	Phase 1	NCT01765426	[121]
Poliovirus	Inactivated virus	Poliomyelitis	Phase 3	NCT03239496	[122]
HIV-1	HIV-1 DNA	AIDS	Phase 2a	PACTR2010050002122368	[123]

^∗^dmLT: double mutant heat-labile enterotoxin.

**Table 4:** Edible and intradermal vaccines: pros and cons.

Route of administration	Host	Pros	Cons
Edible	Plant	Mucosal and systemic immunities, scale-up production, stable transformation, transient transformation, no antigen purification, long-term storage at RT, antigen bioencapsulation, no microbial contaminations	Lack of a proper dosing strategy, improper glycosylation, low antigen expression yields, unstable antigen expression
	Algae	Mucosal and systemic immunities, scale-up production (bioreactors), fast biomass accumulation, easy stable transformation, antigen bioencapsulation, long-term storage at RT	Improper glycosylation, low antigen expression yields
	Insect	Mucosal and systemic immunities, cotranslational modifications, posttranslational modifications, high antigen expression yields, antigen bioencapsulation, stable transformation, transient transformation, high immunogenicity	Improper glycosylation; further studies are needed; cultural barrier
	Yeast	Mucosal and systemic immunities, posttranslational modifications, antigen bioencapsulation, high immunogenicity	Inaccurate glycosylation; further studies are needed
	LAB	Mucosal and systemic immunities, antigen bioencapsulation, high immunogenicity	Possible transfer of antibiotic selection markers

Intradermal		Mucosal and systemic immunities, no systemic side effects, dose sparing, storage at room temperature	Trained personnel for administration, local reactogenicity

LAB: lactic acid bacteria; RT: room temperature.
